# Legitimacy from a Medical and Legal Standpoint.—II

**Published:** 1910-08-27

**Authors:** 


### Medico-Legal Points.

### LEGITIMACY FROM A MEDICAL AND LEGAL STANDPOINT.—II.

### THE GARDNER PEERAGE CASE.

Few trials in relation to legitimacy have excited more attention among jurists than the Gardner Peerage case, which came before the House of Lords in 1825. A full account of the medical evidence was published by Lyall ("Med. Evid. in Gardner Peerage case" 1827). Alan Legge Gardner, the son of Lord Gardner by his second wife, petitioned to have his name inscribed as a peer on the Parliament Roll. The peerage was, however, claimed by another person, Henry Fenton Jadis, *alias* Gardner, who alleged that he was the son of Lord Gardner by his first and subsequently divorced wife. It was contended that the latter son was illegitimate; and in order to establish this point, the evidence adduced was partly medical and partly moral. Lady Gardner, the mother of the alleged illegitimate child, parted from her husband, on board his ship, on January 31, 1802. Lord Gardner went to the West Indies, and did not again see his wife until July 11 following. The child whose legitimacy was disputed was born on December 8 of that year. Therefore, the plain medical question, taking the extreme view, was whether a child born 311 days (forty-four weeks and three days from January to December), or 150 days (twenty-one weeks and three days from July to December) after possible intercourse could be the child of the husband, Lord Gardner. If these questions were answered in the affirmative then it followed that this must have been either a premature or a protracted birth. There was no pretence that it was a premature case, the child having been mature when born. The question then was reduced to this: Was this alleged protracted gestation of 311 days consistent with medical experience? The principal obstetric practitioners in the kingdom were examined on this point. Their evidence was conflicting, but a majority concurred in the opinion that natural gestation might be protracted to a period which would certainly cover the birth of the alleged illegitimate child. On the moral side of the question it was clearly proved that Lady Gardner, after the departure of her husband, was living in open adulterous intercourse with a Mr. Jadis; and on this ground Lord Gardner obtained a divorce from her after his return. He subsequently married a second wife, by whom he had the claimant, Alan Legge Gardner. It was contended that the other claimant was really the son of Lady Gardner by Mr. Jadis. The decision of the House was that this claimant was illegitimate, and that the title should descend to the son of the second Lady Gardner. The decision appears to have been chiefly based on moral circumstances, for had not the first Lady Gardner been living in adultery at the time of her husband's departure, it is highly probable, from the medical evidence bearing strongly that way, that the legitimacy of the child would have been admitted. Again, supposing the child had been born two or three weeks earlier, the question would have resolved itself into this: Who had begotten the child—the husband or the adulterer? This could not have been decided, and then, probably, as in the more recent case of *Anderton* v. *Gibbs*, the rule of law would have pronounced the husband to have been the father. Morally speaking, the decision could not be impugned, but medically speaking it assumed that gestation could never be protracted to the 311th day after probable intercourse. Considering that conception is not necessarily the immediate result of intercourse, and that we have no data for fixing the precise time of its occurrence, this decision could hardly be supported on medical grounds. We should not be justified in affirming that every child born forty-four weeks and three days after the opportunity of intercourse with the husband was *ex necessitate rei* an illegitimate child. Of the seventeen medical experts examined on this occasion, five supported the opinion that the duration of human pregnancy was limited to about nine calendar months, *i.e.* from thirty-nine to forty weeks, or from 273 to 280 days—or, strictly speaking, from 270 to 280 days; one of the witnesses, indeed, said from 265 to 280 days. On the other side, of twelve medical men who seemed to agree respecting the above-mentioned period as the natural term of gestation, the greater number maintained the possibility of pregnancy being protracted to nine and a half, ten, or even eleven calendar months, and, of course, to 311 days—the alleged term of gestation at which the counter-claimant was born—and they thus admitted the possibility that H. F. Jadis *alias* Gardner might be a ten-and-a-half months' child.

### The Guernsey Case.

In *Renouf* v. *Eden* (Queen's Bench, February 1870) an action was brought by a milliner against the defendant for seduction. The plaintiff and defendant met in the island of Guernsey, and it was admitted that an intimacy had existed between them. The defendant left the island on April 15, 1867. and did not return to it. The plaintiff was delivered of a child on February 15, 1868, *i.e.* 307 days, or forty-four weeks minus one day, after the departure of the defendant. There was no evidence that the plaintiff, either before or subsequently, had had connection with any other person. On the part of the defendant, it was contended that he could not possibly have been the father of the child, seeing that, if so, there must have been a period of gestation of over 300 days, which it was contended was physically impossible. Upon this point two physicians were called on each side. Tanner and Clarke, for the plaintiff, declared that though 275 days was the usual period of gestation, they had known cases of 297 or 300 days; and there were in medical books cases of still longer periods. On the other hand,

Tyler, Smith, and Barnes were called, and stated that in their belief the current of medical opinion ran now strongly in an opposite direction, and went to narrow rather than extend the limits of possible gestation; and though they would not go so far as to say that it was absolutely impossible that the period should extend to 300 days, they believed it so improbable as to be practically incredible. On the part of the plaintiff, a case lately decided was quoted to show that even although the child was not the defendant's, yet if he had incited the girl to leave her mother's roof, and then seduced her, the mother was entitled to recover. The Lord Chief Justice agreed in this, and directed the jury that the main issue did not turn upon the medical evidence, for that only went to probabilities, but on all the probabilities of the case. A verdict was returned for the plaintiff, with damages. The medical witnesses on both sides agreed that gestation might be protracted to the extent which would have made the defendant the father of this child.

### Legitimacy Law in Foreign Countries.

The Roman law did not consider an infant legitimate which was born later than ten months after the death of the father, or the dissolution of the marriage. Such was also the French law prior to the Revolution. The Prussian civil code declares that an infant born 302 days after the death of the husband shall be considered legitimate, and a case has occurred, where one born 343 days after the death of the husband was judged a bastard by the legislative commission of that country (Metzger, pp. 427-429). In France and Italy a child born within 180 days after marriage can be repudiated by the husband if no intercourse has taken place between him and his wife before marriage. In Germany the parentage of a child can be repudiated by the husband when he can prove non-intercourse with his wife from the 300th to the 180th day before the birth of the child. An infant born before 180 days after marriage cannot be disowned by him in the following cases: 1. When he had knowledge of his wife's pregnancy before marriage. 2. When he assisted at the act of birth, and signed a declaration of it. 3. When the infant is declared incapable of living.

### The Law in Scotland and the States.

The Scotch law is concise and decisive. "To fix bastardy on a child the husband's absence must continue till within six lunar months of the birth, and a child born after the tenth month is accounted a bastard."

In the case of *The Commonwealth* v. *Porter*, decided in the United States in 1844, the defendant was indicted for fornication and bastardy. The prosecutrix, aged twenty-three, stated that she had intercourse with the defendant on September 24, 1842, and with no other person before or subsequently. She was delivered of a child on August 7, 1843, *i.e.* after 317 days, or forty-five weeks and two days' gestation; and she swore that the defendant was the father of the child. The menses ceased about five weeks after intercourse, and they only appeared again slightly about five weeks before the child was born. At this time she had pains, which continued more or less until her delivery. She first knew that she was pregnant three or four weeks after intercourse. The defence was that, from the period of time which had elapsed, the defendant could not have been the father of the child. He therefore merely proved his absence, and that he did not return until after the birth of the child. No evidence was adduced to impeach the character or conduct of the woman. It was proved that she had always borne a good reputation, and that she had been seduced by the defendant under a promise of marriage. Rodrigue deposed that, in a practice of nineteen years, he had attended some hundreds of cases of midwifery, and the longest period of gestation which he had known was ten months. He considered the pains described by prosecutrix to have been the commencing pains of labour. The court charged the jury strongly in favour of the medical testimony on protracted gestation, and they returned a verdict of guilty, thereby finding that the defendant was the father of the child. It transpired that a wife of one of the jurymen had during one pregnancy gone ten months (*Amer. Jour. Med. Soc.*, October 1845, p. 338).

### Presumption.

In *Evans* v. *Evans and Blyth* (20 T. L. R. 615) Surgeon-Colonel Evans, on June 9, 1903, obtained a decree *nisi* for the dissolution of his marriage with Mary Florence Evans, *née* Dickinson, on the ground of her adultery with Major Blyth. This decree was made absolute on December 21, 1903, and there being (according to the petitioner's view) no living issue of the marriage, he on December 30 filed a petition for variation of settlements. The respondent had, however, on July 31, 1903, given birth to a child, which Major Blyth had on September 6, 1903, registered as his child and that of the respondent, a fact which that lady had on many occasions admitted, both before and since the birth of the child. Accordingly on June 16, 21, 27, 1904, the Court tried the question of the paternity of the child. Mr. Justice Gorell Barnes, in delivering his considered judgment, said that the question he had to decide was whether the child Beatrix Mary was Surgeon-Colonel Evans's child or not, the issues having arisen in the course of an application to vary settlements. The law was quite clear as to the presumption of a child born in wedlock being legitimate. That was the old maxim "*Stabit præsumptio donec probetur in contrarium.*" The leading case in these questions was *Bosville* v. *Attorney-General* (12 P. D. 177), the head-note to which was as follows: "The presumption in favour of the legitimacy of a child born in wedlock is not a '*presumptio juris et de jure,*' but may be rebutted by evidence, which must be clear and conclusive, and not resting merely on a balance of probabilities." In that case Lord Hannen, in his summing up, referred to the judgment of Lord Lyndhurst in *Morris* v. *Davies* (5 Cl. and F. 163), in which he said: "My Lords, this then is the view I have always taken of the law connected with this subject; at the same time, as I before expressed and I now feel, that presumption of law which is that a child born in wedlock is the child of the husband, that presumption of law is not lightly to be repelled. It is not to be broken in upon or shaken by a mere balance of probability, the evidence for the purpose of repelling it must be strong, distinct, satisfactory, and conclusive." Lord Hannen concluded as follows: "It will be for you to say whether the evidence does establish to your mind conclusively, not by way of conjecture but by way of conviction, that the child was not begotten by the husband."

### Some Further Cases.

The verdict of the jury in *Bosville* v. *Attorney-General* was subsequently upheld by the Divisional Court. Mr. Justice Gorell Barnes then critically reviewed the evidence, pointing out that the wife's written confession, dated February 14, 1903, and the fact of the child having been registered on September 16, 1903, by Major Blyth and the respondent was not evidence of the truth of the facts therein referred to, but was evidence of the parties' conduct. To use the words of Mr. Justice North in *Burnaby* v. *Baillie* (42 Ch. D. 282): "I treat the evidence of the statements made by them not as evidence of the truth of the statements which they made, but merely as proving that they did make those statements, thus showing what their conduct was at the time." The difficulties in these cases arose from the old rule of law which prevented parties from themselves giving evidence as to the illegitimacy of their children, although such evidences could be called on their behalf. The broad features of the present case were that the petitioner and the respondent had not been living as husband and wife before the date of her departure for Hagbourne, on or about October 26, 1902, and that Major and Mrs. Blyth had been her guests there until November 4, when Mrs. Blyth only had left. The usual period of gestation was 280 days, although it varied from 270 to 290, and there was a recorded case in the books of 308 days. Taking the period from November 4, 1902, to July 31, 1903, the date of the child's birth, there had elapsed 269 days; but it was to be remembered that in June 1903 the doctor and nurse were engaged by the respondent for her confinement, which she expected about August 12. There was also Major Blyth's admission that he had committed adultery with the respondent at Hagbourne early in November, and that she had joined him after she left her husband in February 1903, and had lived with him since. Further, there was the evidence as to the respondent's state of health during the time that Mrs. Blyth was at Hagbourne. In these circumstances, he found as a fact that the presumption of law had been rebutted, and that Surgeon-Colonel Evans was not the father of the child Beatrix Mary, born on July 31, 1903.

### Hasty Second Marriages.

It might be supposed that common decency, as well as a proper respect for the opinions of mankind, would prevent those sudden marriages which sometimes take place immediately after the death of a former husband. There have, however, been females in all countries who have disregarded these restraints and united themselves to a second partner before the "first brief week of mourning is expired." Besides the injury that such a case produces on the public manners, there is a difficulty which may arise in a legal view. She may be delivered of a child ten months after the death of her first husband, and the question then occurs as to the paternity of the infant.

The Romans endeavoured to prevent this by forbidding the widow to marry until after the expiration of ten months, and this term was prolonged by the Emperors Gratian and Valentinian to twelve. This law has been imitated in the present French code, which also forbids the marriage before ten full months have elapsed since the dissolution of the previous one. The English law on this subject is thus explained by Blackstone and Coke: "If a man dies and his widow soon after marries again, and a child is born within such a time as that by the course of nature it might have been the child of either husband, in this case he is said to be more than ordinary legitimate, for he may, when he arrives at years of discretion, choose which of the fathers he pleases." (Blackstone, vol. i., p. 456. But there is perhaps some doubt about this statement of law.)

